# Real-world effects of Yishen Tongbi decoction for rheumatoid arthritis: protocol for a prospective, observational, multicenter cohort study with validation against double-blind, randomized, controlled trial

**DOI:** 10.3389/fphar.2024.1320578

**Published:** 2024-02-12

**Authors:** Lijuan Liu, Fangfang Zhu, Yijun Xin, Lu Zhang, Congqi Hu, Yanping Xu, Jinming Zhang, Lingjie Liu, Guangxing Chen

**Affiliations:** ^1^ Department of Rheumatology, The First Affiliated Hospital of Guangzhou University of Chinese Medicine, Guangzhou, China; ^2^ Department of Gynecology, Guangdong Provincial Hospital of Chinese Medicine, Guangzhou, China; ^3^ First Clinical Medical School, Guangzhou University of Chinese Medicine, Guangzhou, China; ^4^ Shantou Hospital of Traditional Chinese Medicine, Guangzhou University of Chinese Medicine, Shantou, China; ^5^ Baiyun Hospital of The First Affiliated Hospital of Guangzhou University of Chinese Medicine, Guangzhou, China

**Keywords:** rheumatoid arthritis, Yishen Tongbi decoction, methotrexate, real-world study, protocol

## Abstract

**Introduction:** Rheumatoid arthritis (RA) is a globally challenging and refractory autoimmune disease, constituting a serious menace to human health. RA is characterized by recurrent pain and is difficult to resolve, necessitating prolonged medication for control. Yishen Tongbi decoction is a traditional Chinese herbal compound prescribed for treating RA. We have completed a 3-year RCT study that confirmed the clinical efficacy of Yishen Tongbi decoction for RA. Notably, we observed a faster clinical remission rate compared to MTX by week 4 of treatment. In our forthcoming study, we intend to conduct a comprehensive assessment of the efficacy and safety of Yishen Tongbi decoction in the real-world treatment of RA through a prospective study.

**Methods and analysis:** This prospective, multicenter, real-world observational study will be conducted at two designated centers in China from October 2023 to August 2025. The study will include 324 patients with active rheumatoid arthritis. One group will receive Yishen Tongbi decoction combined with conventional synthetic disease-modifying antirheumatic drugs (csDMARDs). The other group will receive standard treatment. Standard treatment can be further divided into subgroups: csDMARDs, targeted synthetic disease-modifying antirheumatic drugs (tsDMARDs), and biologic disease-modifying antirheumatic drugs (bDMARDs). In each group, the number of tender joints, number of swollen joints, pain score, patient global assessment, physician global assessment, disease activity index (DAS28-ESR or DAS28-CRP), clinical disease activity index (cDAI), simplified disease activity index (sDAI) and relevant laboratory data will be compared. Clinical indicators and disease activity of the patients will be assessed at baseline, week 4 and week 12 after the initiation of treatment. The primary outcome will be the American College of Rheumatology 20% improvement criteria (ACR20) attainment rate among patients at week 12 after treatment. Every adverse event will be reported.

**Ethics and dissemination:** This study has been approved by the Ethics Committee of the first affiliated Hospital of Guangzhou University of traditional Chinese Medicine (NO.K-2023-009). The results of the study will be published in national and international peer-reviewed journals and at scientific conferences. The researchers will inform participants and other RA patients of the results through health education.

**Clinical Trial Registration:** https://www.chictr.org.cn/index.html, identifier ChiCTR2300076073

## Introduction

Rheumatoid arthritis (RA) is a systemic inflammatory autoimmune disease. Patients often exhibit joint pain and swelling, and disease progression can result in joint deformities and functional loss, significantly impairing physical function and quality of life (Smolen et al., 2018). It affects approximately 0.5%–1% of the global population, with an 8.2% increase in incidence between 1990 and 2017 (Gary et al., 2001; Finckh et al., 2022). As a lifelong condition, RA has made remarkable progress in the study of its pathogenesis and pathological changes in recent years, alongside an expanded array of available medication choices (Smolen et al., 2023).

The clinical management of RA necessitates personalized drug selection, giving rise to a diverse array of treatment approaches and primary medications. Conventional synthetic disease-modifying antirheumatic drugs (csDMARDs) is first-line treatment for RA, with methotrexate (MTX) being the current standard treatment for RA. However, it usually takes 6–8 weeks or even longer to observe clinical improvement (Wang et al., 2018). The clinical advancement and utilization of biologic disease-modifying antirheumatic drugs (bDMARDs) and targeted synthetic disease-modifying antirheumatic drugs (tsDMARDs) introduce fresh prospects in the treatment of RA. However, the infection and tumor risks associated with their prolonged utilization require further assessment (Burmester et al., 2015; Tanaka, 2023). In recent years, several randomized controlled trials (RCTs) have indicated that combining traditional Chinese herbal medicine with Western medicine is superior to Western medicine alone, providing both better outcomes and fewer adverse reactions (Gong et al., 2021; Shen et al., 2021).

Yishen Tongbi decoction (YSTB) is an herbal formula consisting of six medicinal herbs, including *Tripterygium wilfordii* Hook.f [Celastraceae; *T. wilfordii radix et rhizoma*] (Kunming Shan Hai Tang) (THH), *Eucommia ulmoides* Oliv. [Eucommiaceae; *Eucommiae Cortex*] (Du Zhong), *Ligustrum lucidum* W.T.Aiton [Oleaceae; *Ligustri Lucidi Fructus*] (Nv Zhen Zi), *Eclipta prostrata* (L.) L. [Asteraceae; *Ecliptae Herba*] (Han Lian Cao), *Lycium barbarum* L. [Solanaceae; *Lycii Fructus*] (Gou Qi), and *Salvia miltiorrhiza* Bunge [Lamiaceae; *Salviae miltiorrhizae radix et rhizoma*] (Dan Shen). We analyzed the primary chemical constituents in the extracts and evaluated the quality of YSTB extracts using UPLC-Q-TOF-MS/MS (The chromatogram of YSTB is presented in Supplementary Material S1). It is protected by Patent No. ZL 2010110191532. X and has shown efficacy in treating RA over a decade of use. We have completed a 24-week double-blind, double-model, randomized controlled trial comparing the effectiveness and safety of YSTB with MTX for active RA. At week 4, the YSTB groups exhibited a significantly higher attainment of ACR20 and EULAR good or moderate responses, indicative of a more rapid therapeutic effect compared to MTX (Xu et al., 2023). In our animal experiments, we observed that YSTB possesses anti-inflammatory properties, inhibits synovial hyperplasia, and improves bone destruction (Hu et al., 2021; Jiao et al., 2023). Cell experiments revealed that YSTB could inhibit the activation and proliferation of ST486 cells (Burkitt’s lymphoma ST486 human B cells) and promote the apoptosis of ST486 cells (Hu et al., 2021). The Main components of YSTB is shown in Table 1.

**TABLE 1:** Composition and action of Yishen Tongbi decoction.

Pinyin name	Full species name	Pharmacological effects	Toxicity	Doses (g)
Kunmingshanhaitang	*Tripterygium wilfordii* Hook.f. [Celastraceae; *Tripterygium wilfordii radix et rhizoma*]	Kunmingshanhaitang has strong anti-inflammatory and immunosuppressive effects, which can significantly improve joint swelling and pain and delay bone destruction in patients with RA Chatzidionysiou et al. (2017), Drosos et al. (2020), Yates et al. (2020), Fujii et al. (2024)	hepatotoxicity, nephrotoxicity, reproductive toxicity Ru et al. (2019)	25
Nvzhenzi	*Ligustrum lucidum* W.T.Aiton [Oleaceae; *Ligustri Lucidi Fructus*]	Nvzhenzi can improve bone metabolism and inhibit osteoclast differentiation so as to achieve the effect of anti-osteoporosis Du (2010)	NA Cao et al. (2023)	15
Duzhong	*Eucommia ulmoides* Oliv. [Eucommiaceae; *Eucommiae Cortex*]	Duzhong can inhibit the secretion of pro-inflammatory cytokines, promote the secretion of anti-inflammatory cytokines, inhibit the proliferation of synovial cells and delay the degradation of chondrocytes and bones Wei et al. (2014)	NA Huang et al. (2021)	15
Gouqi	*Lycium barbarum* L. [Solanaceae; *Lycii Fructus*]	Gouqi has immunomodulatory, anti-inflammatory and anti-osteoporotic effects in the treatment of RA Chen et al. (2018)	NA Yu et al. (2023)	15
Danshen	*Salvia miltiorrhiza* Bunge [Lamiaceae; *Salviae miltiorrhizae radix et rhizoma*]	Danshen has specific anti-inflammatory and anti-immune effects Zhao et al. (2014)	NA Meim et al. (2019)	15
Hanliancao	*Eclipta prostrata* (L.) L. [Asteraceae; *Ecliptae Herba*]	Hanliancao can inhibit the proliferation and differentiation of RAW264.7 osteoclasts Xie et al. (2015)	NA Timalsina and Devkota (2021)	15

The decoction was made in the following manner: (1) Mix an appropriate quantity of cold water with Tripterygium wilfordii Hook.f. (THH; 25 g) and an equivalent amount of cold water with Ligustrum lucidum W.T.Aiton (15 g), Eclipta prostrata (L.) L. (15 g), Eucommia ulmoides Oliv. (15 g), Lycium barbarum L. (15 g), and Salvia miltiorrhiza Bunge (15 g) in a casserole, allowing it to steep for 30 min. (2) Boil THH, in 10 volumes of water for 3.5 h. (3) Combine the infusion of the remaining five herbs with 8 volumes of water, adding it to the THH, decoction that has boiled for 3.5 h, and simmer them together for an additional 40 min. (4) Strain out the residue and consume 150 mL once a day, preferably warm after meals.

We intend to validate these findings in extensive real-world clinical practice, recognizing the greater complexity of RA treatment in real-life scenarios compared to clinical trials. For these reasons, we aim to conduct a prospective real-world observational study with more subgroup analysis that focuses not only on the impact of YSTB on clinical outcomes, but also on the comparison of different treatment schemes in the real world.

### Objective

Based on previous RCT studies on YSTB, we will initiate a prospective real-world study to comprehensively assess the efficacy and safety of YSTB in RA treatment. Subgroup analyses will be used to evaluate the clinical remission effectiveness in RA patients receiving various treatment strategies. Furthermore, we will assess whether the combination of YSTB can provide additional significant beneficial results on the basis of csDMARDs.

## Methods and analysis

### Study design

**FIGURE 1:**
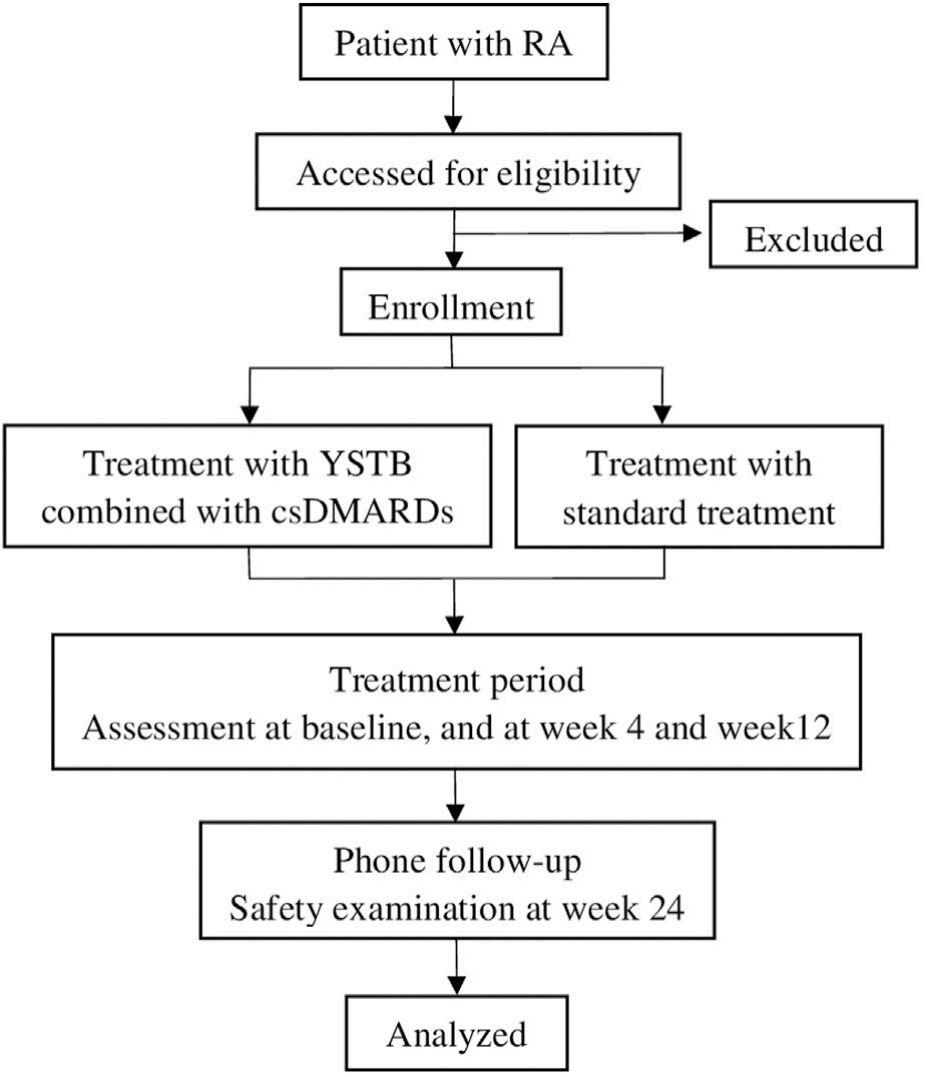
Flow diagram of the clinical trial. RA Rheumatoid arthritis, YSTB Yishen Tongbi decoction, standard treatment includes bDMARDs/tsDMARDs along with csDMARDs, or a combination of two csDMARDs.

**FIGURE 2:**
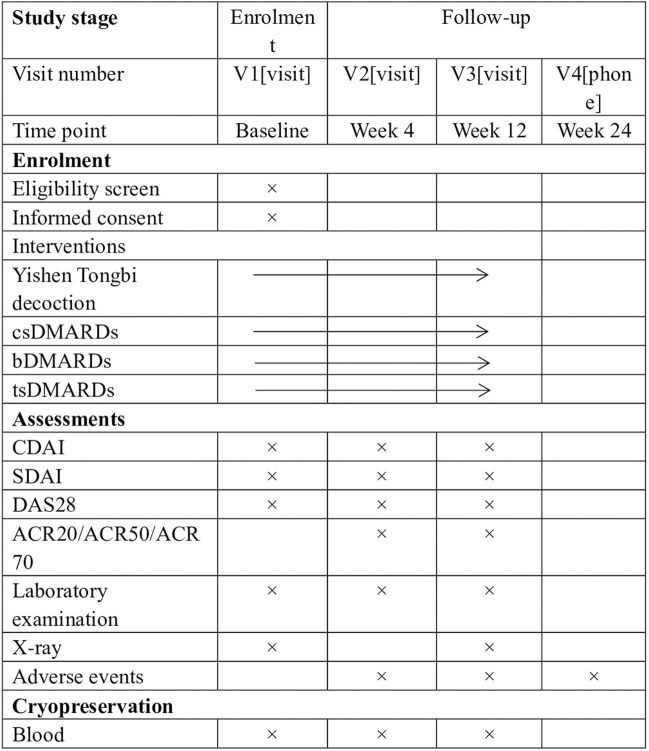
SPIRIT figure showing time points for enrolment, interventions and assessment. *Laboratory examination includes complete blood count, rheumatoid factor, Anticyclic citrullinated peptide antibody, C-reactive protein, erythrocyte sedimentation rate, liver function tests (aspartate aminotransferase, alanine aminotransferase) and renal function (creatinine, urea). csDMARDs Conventional synthetic disease-modifying antirheumatic drugs, bDMARDs Biological disease-modifying antirheumatic drugs, tsDMARDs Targeted synthetic disease-modifying antirheumatic drugs, ACR20/50/70 improvement in American College of Rheumatology criteria of 20%/50%/70%, CDAI Clinical Disease Activity Index, DAS28 Disease Activity Score in 28 joints, MTX methotrexate, SDAI Simplified Disease Activity Index.

This study is a prospective, multicenter, real-world observational clinical trial conducted at two designated centers in China from October 2023 to August 2025. The study included a population of 324 patients with RA. Treatment schemes were determined by doctors based on individual patient conditions and preferences. One group will receive YSTB combined with csDMARDs, while the other group will receive bDMARDs/tsDMARDs combined with csDMARDs, or a combination of two csDMARDs. The study protocol has been approved by the Ethics Committee of the First Affiliated Hospital of Guangzhou University of Traditional Chinese Medicine (approval NO.K-2023-009) and registered with the Chinese Clinical Trial Registry (ChiCTR2300076073). The study flowchart is shown in Figure 1. The Standard Protocol Items: Recommendations for Interventional Trials (SPIRIT) figure is shown in Figure 2.

### Inclusion criteria

Subjects must meet all of the following requirements:

1) Meet the 2010 American College of Rheumatology (ACR)/European League against Rheumatism classification criteria and have RA for at least 6 weeks
2) Have received and maintained standard RA treatment for at least 4 weeks
3) Assessed as patients with mild, moderate, or severe RA based on DAS28-ESR/DAS28-CRP criteria
4) Provide signed informed consent form

### Exclusion criteria

Subject who does not meet any of the following will be excluded:

1) Pregnant or lactating women
2) Individuals with current or future fertility concerns
3) Currently using Chinese patent medicine or traditional Chinese medicine decoction that bear similarities to the experimental drugs in terms of their constituent components
4) History of chronic severe infection, any current infection and any malignant tumor
5) Patients with severe primary diseases, such as those of the cardiovascular, cerebrovascular, hepatic, renal, and hematopoietic systems, and mental illness

### Sample size estimation

Based on previous study and physician experience (Xu et al., 2023), this study comprises two independent trials with comparative rates. Its objective is to explore whether there exists a disparity in the ACR20 response rate between the two groups after 12 weeks of treatment. The prior study conducted by our research group indicated that after 12 weeks of treatment, the ACR20 response rate was 87% for the YSTB combined with the MTX group, while it was 74% for the MTX group. Assuming *α* = 0.05 (two-tailed test) and achieving a power of (1-β) = 0.8, we utilized PASS 15 software to calculate sample sizes for the experimental and control groups, totaling 324 patients to be included.

### Interventions

This observational study is to evaluate the efficacy and safety of YSTB in the real-world hospital setting. Therefore, for the enrolled participants, there is no strictly defined treatment regimen. After evaluating the patient’s condition, the doctor chooses the most appropriate therapeutic medication treatment medication. Patients who receive YSTB (150 mL orally once a day) combined with csDMARDs to manage their condition will be divided into YSTB combined with csDMARDs group. Patients who are treated with bDMARDs/tsDMARDs, along with csDMARDs, or those using a combination of two csDMARDs, will be placed in the standard treatment group. As for specific drugs, common csDMARDs may include medications like Methotrexate, Leflunomide, or Apremilast, while tsDMARDs could consist of drugs such as Tofacitinib tablets or Baricitinib tablets. bDMARDs may include medications like Yisaipu. Nonsteroidal anti-inflammatory drugs (NSAIDs) or oral corticosteroids (prednisone ≤10 mg/days or the same dose) can be added when necessary. The medication information of the participants will be recorded in the case report form (CRF) at each follow-up.

### Outcome measures

The researchers will collect clinical data from patients at baseline, as well as at week 4 and week 12 following the initiation of treatment. Every Adverse event will be reported in time. The primary outcome is the attainment rate of the American College of Rheumatology 20% improvement criteria (ACR20) attainment rate among patients at week 12. ACR20 includes the number of tender joints, the number of swollen joints, and the fulfillment of at least 3 out of the following 5 criteria: visual analog scale (VAS) for Pain, patient global assessment, physician global assessment, Health Assessment Questionnaire (HAQ), C-reactive protein (CRP) or erythrocyte sedimentation rate (ESR). The above observation indicators meet the proportion of patients whose improvement reached 20%.

The secondary outcome is at week 12, the percentage of patients achieving clinical remission (DAS28-ESR <2.6) or low disease activity (LDA,DAS28-ESR <3.2) with the combination of YSTB combined with csDMARDs. The proportion of patients achieving a ≥50% improvement in cDAI or cDAI ≤2.8; the proportion of patients achieving a ≥50% improvement in sDAI or sDAI ≤3.3; The proportion of patients achieving ACR70, ACR50, EULAR good or moderate response, and a normal Health Assessment Questionnaire Disability Index score (≤0.5); Cumulative duration of effective treatment during the double-blind period; Results of patients’ self-evaluation. Adverse event is defined as all symptoms, signs, disease or discomfort that occur or aggravate during the follow-up process, including abnormal liver function, leukopenia, gastrointestinal reactions, rashes, palpitations, *etc.*

### Timeline of visits

The study follow-up period is expected to be 12 months. According to published literature and relevant clinical practice experience, the patient is expected to complete the following follow-up visits:

Pre-enrollment visit: Patients who provided signed informed consent are assessed with regard to personal data and complete clinical history, including diagnostic confirmation and demographic information.

Follow-up: At baseline, week 4, and week 12, we collect data on patients’ affected joint status, morning stiffness and pain assessments, laboratory tests, disease activity assessments, and health assessment questionnaires. General data collection occurs during the baseline visit, and X-rays of the dual-hand joints are obtained both at baseline and at week 12.

### Data collection

We designed a standardized Case Report Form (CRF) for data collection, including demographics, joint condition, pain and stiffness assessments, laboratory results, disease activity, health questionnaires, X-ray, and outcomes. Investigators ensure accurate CRF and medical record completion. Any incorrect data or text should not be altered but should be marked with a single line, and re-filled in the correct data and text on the side, as well as the researcher’s signature and current date. CRF data will be entered into the ResMan database using two methods, cross-verified for accuracy. Original medical records remain at each center, and all research-related data will be kept in the Department of Rheumatology, the First Affiliated Hospital of Guangzhou University of TCM. The data will be processed anonymously, omitting information that can identify the participant. The archives of clinical trial institutions will establish strict security and confidentiality measures.

### Adverse events recording, evaluation, analysis, and reporting

An adverse event (AE) is the presence or worsening of any syndrome, symptom or disease that occurs in a patient during the clinical trial. For each adverse event, the researchers will carefully record the start date, end date, degree, and relationship with the trial, and follow up all adverse events until they are properly resolved or the patient’s condition is stable. Every adverse event will be submitted to the sponsors, ethics committee, and drug regulatory authorities as required.

Serious AEs are events that result in hospitalization, prolonged hospital stays, disability (excluding joint damage from rheumatoid arthritis), inability to work (excluding joint damage from rheumatoid arthritis), or are life-threatening or fatal during the clinical trial. The researcher will immediately inform the principal investigator of any serious AEs, in accordance with the study plan and standard operating procedures, and report them to the Good Clinical Practice office and the relevant department within 24 h. Adequate compensation will be provided to affected subjects.

### Statistical analyses

Attempting to ensure equivalence in baseline characteristics of participants between the YSTB group and the conventional therapy group, a propensity score method will be performed. Variables involving age, sex, smoking history, BMI and other factors will be included into the logistic regression model to calculate a propensity score for each participant. Participants who used YSTB will be matched to those who receive conventional drugs alone based on the propensity score, using the greedy matching with a standard calliper width of 0.2 and without case replacement. Covariate balance of the matched cohort will be assessed using the mean standardized differences. When a |d| > 0.10, it is considered as an imbalance. Inverse probability of treatment weighting based on the propensity score will also be performed to evaluate the robustness of the matching results. Baseline demographic variables will be presented as means, medians, or percentages, and using Chi-square test, independent *t*-test, or Mann-Whitney *U* test, as appropriate. Outcomes of CDAI, SDAI, DAS-28, ACR20, ACR50, ACR70 will be analyzed by using Chi-square test and logistic regression. Odd ratios and 95% confidence intervals will be estimated. Subgroup analysis will be performed on age, gender, treatment regimens (CHM alone, conventional medication alone and the combination). All analysis will be tested with a two-sided alpha level of 0.05 except the logistic regression. For the missing data, the reasons why the data is missing will be analyzed, and multiple imputation will be used.

## Discussion

There are three clinical problems in RA that need to be solved urgently: 1) the pathogenesis is complex, and it is difficult to diagnose and treat early. 2) lack of high-efficient and low-toxic drugs, and the therapeutic mechanism of traditional Chinese medicine is unknown. 3) the therapeutic advantages of traditional Chinese medicine lack of high-quality research evidence. Therefore, on the basis of explaining the pathogenesis and core pathological targets of RA, it is very necessary and urgent to establish new strategies for the treatment of traditional Chinese medicine and develop new drugs with high efficiency and low toxicity.

YSTB is a patented Traditional Chinese Medicine (TCM) formulation used for the treatment of RA for over a decade. Previous RCT studies have shown that YSTB has a quick effect, which is not inferior to methotrexate, and has fewer side effects. Current standard RA treatments include bDMARDs/tsDMARDs along with csDMARDs, or a combination of two csDMARDs. The combined use of csDMARDs may cause bone marrow suppression and hepatorenal toxicity (Drosos et al., 2020; Yates et al., 2020). The application of tsDMARDs in individuals with low immunity, particularly the elderly, can lead to herpes zoster infections and increased thrombotic risks (Fujii et al., 2024). The high cost of bDMARDs restricts their use and may activate latent infections like tuberculosis or hepatitis, as well as increase the risk of tumor development (Chatzidionysiou et al., 2017). In light of these issues, we embarked on a real-world study to develop safe, effective TCM compounds with minimal side effects. The aim is to establish YSTB in combination with csDMARDs as a treatment option that is not inferior to standard treatments and offers superior safety and cost-effectiveness.

The theory of traditional Chinese medicine and the practice of doctors of previous dynasties have shown that the cause of RA is the deficiency of liver and kidney, exogenous wind, cold, dampness and heat, which finally leads to the obstruction of meridians. Combined with the theory of traditional Chinese medicine and long-term clinical practice, our research group developed “Yishen Tongbi decoction” for the treatment of RA with “Bushen Tongluo” as the specific treatment. The prescription is composed of *L. lucidum* W.T.Aiton 15 g, *E. prostrata* (L.) L. 15 g, *E. ulmoides* Oliv. 15 g, *L. barbarum* L. 15 g, *S. miltiorrhiza* Bunge 15 g, and *T. wilfordii* Hook. f. 25 g. Combined with the contemporary theory of “medicine group” (Du, 2010), the four groups of *L. lucidum* W.T.Aiton, *E. prostrata* (L.) L., *E. ulmoides* Oliv. and *L. barbarum* L. were used to tonify the liver and kidney and strengthen the muscles and bones. According to the principle of “treating as a king and treating as a minister” (Wei et al., 2014), *S. miltiorrhiza* Bunge mainly promotes blood circulation and removes blood stasis, relieves menstruation and relieves pain, *T. wilfordii* Hook. f. mainly dispels wind and dehumidification, relaxes muscles and activating collaterals, the above two flavors are subject medicine group, take its blood circulation and dredge collaterals to relieve pain. The party and its preparation process have been authorized by the national invention patent (ZL2010110191532.X). *Tripterygium wilfordii* Hook.f. has been proved to have strong anti-inflammatory and immunosuppressive effects by many clinical and basic studies, which can significantly improve joint swelling and pain and delay bone destruction in patients with RA (Zhang et al., 2007; Zhao et al., 2014; Xie et al., 2015; Chen et al., 2018). *Ligustrum lucidum* W.T.Aiton and its extract can improve bone metabolism and inhibit osteoclast differentiation so as to achieve the effect of anti-osteoporosis (Chen et al., 2017). The ethanol extract of *E. ulmoides* Oliv. can inhibit the secretion of pro-inflammatory cytokines, promote the secretion of anti-inflammatory cytokines, inhibit the proliferation of synovial cells and delay the degradation of chondrocytes and bones, all of which can improve the symptoms of RA (Wang et al., 2016). *Lycium barbarum* L. has immunomodulatory, anti-inflammatory and anti-osteoporotic effects in the treatment of RA (Cheng et al., 2015). Studies have shown that *S. miltiorrhiza* Bunge has specific anti-inflammatory and anti-immune effects, which can significantly improve the related symptoms of mice (Gao et al., 2012). *Eclipta prostrata* (L.) L. and its active components inhibit the proliferation and differentiation of RAW264.7 osteoclasts at low doses. These results suggest that *E. prostrata* (L.) L. may be used in the treatment of osteoporosis (Liu et al., 2014).

Traditional Chinese medicine theory, clinical practice and many basic studies have shown that Yishen Tongbi decoction has a significant effect in the treatment of RA, but the therapeutic advantage of traditional Chinese medicine is still lack of high-quality evidence-based medicine. Therefore, the research group conducted a double-blind, randomized, controlled, non-inferiority trial of YSTB in the treatment of active RA, head-to-head comparison of the efficacy and adverse reactions of MTX (Xu et al., 2023). This clinical trial is the first RCT study in China to use traditional Chinese medicine compound prescription and MTX to conduct a head-to-head study in the field of RA treatment. This study uses CDAI as the main outcome index for a period of 24 weeks. The results showed that the standard rate of CDAI in YSTB group and MTX group were 67.4% (33/49) and 57.1% (28/49), respectively, and the risk difference was 0.102 (95% CI −0.089 to 0.293), indicating that Yishen Tongbi decoction was not inferior to MTX. In the secondary outcome index, compared with the MTX group, the YSTB group had a higher rate of good or moderate response to ACR20 (57.1% vs. 30.6%, *p* = 0.008) and good or moderate response to EULAR (61.2% vs. 34.7%, *p* = 0.009) at week 4, suggesting that YSTB took effect faster than MTX. The incidence of drug-related adverse events was 28.6% (14/49) and 22.4% (11/49) in the YSTB and MTX groups, respectively. There was no significant difference in the incidence of drug-related adverse reactions between the two groups (*p* = 0.487), and there was no serious non-reaction, which proved the safety of YSTB. Both YSTB and MTX groups showed impaired liver function (ALT >33 U/L) as the most frequent drug-related side effect, with rates of 14.3% (7/49) and 12.2% (6/49) respectively (*p* = 0.766). Although liver function abnormalities were more common than other adverse events, 4 patients in each group had slightly elevated levels (<2 times the upper limit), mostly asymptomatic, allowing trial completion. One YSTB patient with ALT of 123 U/L at week 12 normalized with outpatient liver protection. Gastrointestinal reactions occurred in 2.0% (1/49) in both groups (*p* = 1.000). Diarrhea led to study discontinuation in one patient per group, and one YSTB patient stopped due to paroxysmal palpitations. Leukopenia incidence was 2.0% in both groups, and one MTX patient appeared anemic. Menstrual irregularities affected 3.5% (3/86) of female patients (43 premenopausal) without significant group difference (*p* = 0.554) during the 24-week trial (Xu et al., 2023). This study provides high-quality evidence of evidence-based medicine for the treatment of RA with YSTB, and the relevant results are published in the first District Journal of the highest influencing factor in the field of traditional Chinese medicine.

Although RCT is the gold standard for evaluating the safety and effectiveness of interventions, it has some limitations, such as strict inclusion and exclusion criteria, relatively small sample size, poor external authenticity, and high difficulty and cost of implementation. Real-world study (RWS) is a continuation and supplement of RCT, which aims to evaluate the actual efficacy of interventions in the real diagnosis and treatment environment under a large sample size, emphasizing external authenticity, and is widely used in disease diagnosis and treatment, prognosis and etiological analysis. High-quality RWS can accelerate the approval of new drugs and new indications (Knottnerus and Tugwell, 2010) In addition, the previous RCT study is a single-center study, a total of 82 patients completed the trial, the sample size is small, the multicenter trial with larger sample size is needed to obtain more objective clinical data for verification. Based on the above two points, our research group plans to carry out real-world study on the treatment of active RA patients with YSTB, in order to provide high-quality evidence-based medicine for the treatment of RA with traditional Chinese medicine compound, and promote the research and development of new drugs with low toxicity and high efficiency against RA.

Our research also has some limitations. First of all, there are subjective indicators evaluated by patients and doctors in the study. Therefore, these results may be biased. We can only assume that doctors maintain relatively uniform evaluation criteria and patients need further guidance on how to correctly assess the disease. Secondly, due to various reasons, it may be difficult for patients to adhere to the 12-week study cycle, so it is necessary to strengthen follow-up and timely communication with patients to minimize the drop-out rate. After the completion of the trial, we hope to make a clear conclusion on the efficacy and safety of YSTB.

Drawing upon past clinical experience and RCT studies, we hypothesize that YSTB combined with csDMARDs is not inferior in efficacy to the standard treatment group, offers better safety, and is more cost-effective. This research aims to offer more treatment options for RA patients. We have conducted RCT research on the treatment of RA with YSTB compound formulas and are planning to carry out a real-world study, aiming to provide more comprehensive data on YSTB. In the future, we intend to design comparative studies between YSTB decoction and granules, as well as develop hospital preparation and new drug launches, aiming to benefit more RA patients with YSTB.

## Data Availability

The original contributions presented in the study are included in the article/Supplementary Material, further inquiries can be directed to the corresponding author.

## Abbreviations

YSTB, Yishen Tongbi decoction; RA, rheumatoid arthritis; MTX, methotrexate; RCTs, randomized controlled trials; csDMARDs, conventional synthetic disease-modifying antirheumatic drugs; bDMARDs, biologic disease-modifying antirheumatic drugs; tsDMARDs, targeted synthetic disease-modifying antirheumatic drugs; NSAIDs, Nonsteroidal anti-inflammatory drugs; ACR20, American College of Rheumatology 20% improvement criteria.
