# Supplementary material for: Real-world effects of Yishen Tongbi decoction for rheumatoid arthritis: protocol for a prospective, observational, multicenter cohort study with validation against double-blind, randomized, controlled trial
Source: Front Pharmacol. 2024 Feb 12;15:1320578. doi: 10.3389/fphar.2024.1320578 (PMC10895057; doi:10.3389/fphar.2024.1320578)
Supplement: Supplementary file 1 [file DataSheet2.PDF]

Analysis of Chemical constituents of Yishen Tongbi decoction based on UPLC-Q-TOF/MS

1. Materials

ExionLC AC Liquid chromatograph、X500R QTOF Mass spectrometer（Sciex Corporation of USA）；Mettler TOLEDO Electronic Analytical balance (Switzerland)；KQ-700DE numerical control ultrasonic cleaning instrument (Kunshan Ultrasonic instrument Co., Ltd.).

2.Method

2.1 Sample preparation

Take the sample about 5mL, put it in a 50 mL conical bottle with plug, ultrasonic (power 300W, frequency 40 KHz), treat it for 30 min, cool, shake well, centrifuge (13000 r/min) for 10 min, and get the supernatant.

2.2 Chromatography

UPLC-Q-TOF-MS/MS analysis was performed on an Acquity BEH C_{18} column (100 × 2.1 mm, 1.7 μm, Waters, MA, USA). The mobile phase consisted of 0.1 % formic acid aqueous solution (A) and acetonitrile (B): 0~8 min, 3 %~15 % B; 8~16 min, 15 %~28 % B; 16~25 min, 28 %~50 % B; 25~36 min, 50 %~85 % B; and 36~38 min, 85 %~100 % B. The column temperature was maintained at 40 °C. The flow rate was 0.30 mL/min, and the injection volume was 1.0 μL.

2.3 Mass spectrometry

Chromatograms were acquired using ESI in both positive and negative ion mode. The Q-TOF-MS/MS settings were as follows: ion source gas 1 and gas 2, both 50 psi; curtain gas, 35 psi; ion source temperature, 500 °C; ion-spray voltage, +5500/− 4500 V; declustering potential voltage, 100/− 80 V; collision energy, ± 35 V; and collision energy spread, 15 V. Samples were analyzed in both positive and negative ionization modes with a scanning mass-to-charge (m/z) range of 100–1000.

3.Results and analysis

3.1 Identification result

According to the high-resolution mass spectrometry data to analyze the retention time, accurate relative molecular weight and fragment ion information of the compounds, and combined with the comparison of the control substance and the literature, there were 37 compounds

identified in YSTB. Details are provided in Table 1.

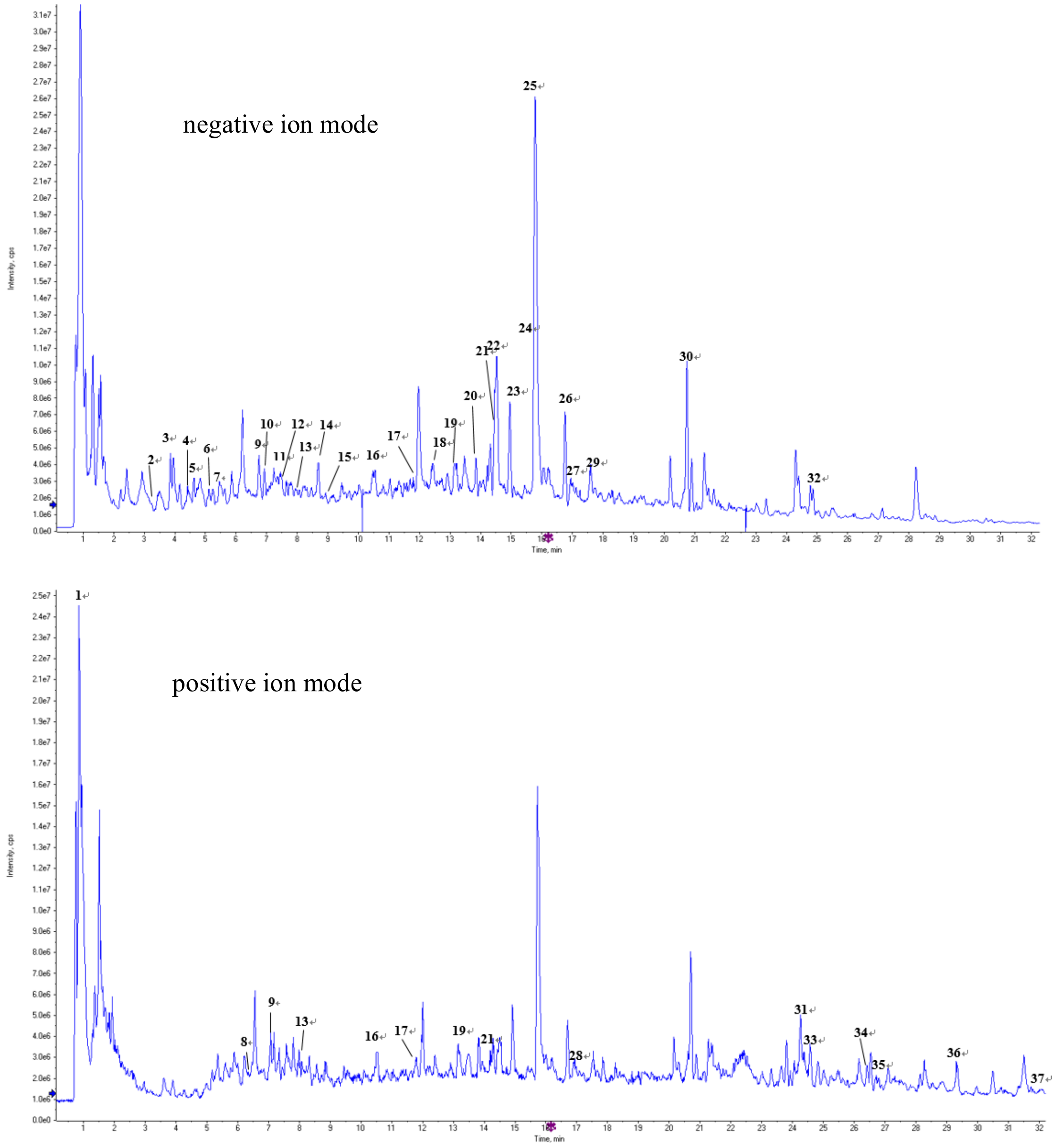

Fig.1. Total ion flow diagram of Yishen Tongbi decoction

Table 1 results of UPLC-Q-TOF/MS identification and analysis of chemical constituents in

Yishen Tongbi decoction

NO	Rt(min)	Proposed compound	Formula	Positive ion (m/z)			Negative ion(m/z)		
				indicated	ppm	Fragment	indicated	ppm	Fragmen
1	0.867	betaine [1]a	$C_5H_{11}NO_2$	118.0859	-3.1	118.0861, 58.0649	-	-	-
2	3.264	aucubin[2]	$C_{15}H_{22}O_9$	-	-	-	391.1242	-1	183.0661, 165.0562,1
3	3.854	danshensu [3]	$C_9H_{10}O_5$	-	-	-	197.0454	-1	179.0352,135.0450,1
4	4.430	hydroxytyrosol[4]a	$C_8H_{10}O_3$	-	-	-	153.0556	-0.9	123.0454
5	4.625	geniposidic acid[2]a	$C_{16}H_{22}O_{10}$	-	-	-	373.1137	-0.9	211.0611,167.0712,1
6	5.133	neochlorogenic acid[2]	$C_{16}H_{18}O_9$	-	-	-	353.0875	-1	191.0559,179.0349,1
7	5.538	3,4-dihydroxybenzaldehyde[3][5]	$C_7H_6O_3$	-	-	-	137.0244	0	137.0244,108.0215
8	6.361	salidroside[4][6]a	$C_{14}H_{20}O_7$	318.1548	0.2	145.0493,121.0649,69.0336	-	-	-
9	6.760	epigallocatechin [5]a	$C_{15}H_{14}O_6$	291.0860	-1.2	147.0440,139.0388,123.0440	289.0715	-0.9	245.0816,203.0711,1
10	6.935	chlorogenic acid[2][7]	$C_{16}H_{18}O_9$	-	-	-	353.0875	-1	191.0561,85.0294
11	7.367	caffeic acid[2][3][5]	$C_9H_8O_4$	-	-	-	179.0348	-1	135.0454
12	7.4534	cryptochlorogenic acid[2]	$C_{16}H_{18}O_9$	-	-	-	353.0875	-0.8	19.0561,179.0350,13
13	7.982	procyanidin B2[11]a	$C_{30}H_{26}O_{12}$	579.1493	-0.8	427.1031,291.0864	577.1346	-0.9	451.1035,407.0772,2
14	8.696	(-)-epicatechin[5]	$C_{15}H_{14}O_6$	-	-	-	289.0716	-0.7	245.0819,203.0714,1
15	8.941	geniposide[2]a	$C_{17}H_{24}O_{10}$	-	-	-	433.1347	-1	225.0766
16	10.554	pinoresinol diglucoside [2]	$C_{32}H_{42}O_{16}$	700.2815	0.5	341.1386,235.0967,175.0750	681.2395	-0.7	51.1877,357.1347,15
17	11.814	rutin[2]	$C_{27}H_{30}O_{16}$	611.1609	0.3	303.0498	609.1455	-1	301.0349
18	12.41	cynaroside [7]	$C_{21}H_{20}O_{11}$	-	-	-	447.0928	-1	285.0403

NO	Rt(min)	Proposed compound	Formula	Positive ion (m/z)			Negative ion(m/z)		
				indicated	ppm	Fragment	indicated	ppm	Fragmen
19	13.228	3,5-dicaffeoylquinic acid [2]	$C_{25}H_{24}O_{12}$	517.1339	-0.3	163.0387,145.0284	515.1191	-0.8	353.0871,191.0558,1
20	13.856	specnuezhenide[4][6-7]a	$C_{31}H_{42}O_{17}$	-	-	-	685.2343	-1	523.1822,453.1398,4
21	14.443	4,5-di-O-caffeoylquinic acid [2]	$C_{25}H_{24}O_{12}$	517.1339	-0.3	163.0388	515.1190	-1	353.0876,179.0351,1
22	14.538	rosmarinic acid[3][8]	$C_{18}H_{16}O_8$	-	-	-	359.0769	-1	197.0453,179.0347,1
23	14.953	isosvianolic acid A [3]	$C_{26}H_{22}O_{10}$	-	-	-	493.1137	-0.7	313.0716,295.0608,1
24	15.721	oleuropein [4][6-7]a	$C_{25}H_{32}O_{13}$	-	-	-	539.1767	-0.6	377.1243,307.0821,1
25	15.790	salvianolic acid B[3][8]a	$C_{36}H_{30}O_{16}$	-	-	-	717.145	-1.5	519.0945,339.0499,3
26	16.761	salvianolic acid E[3][8]	$C_{36}H_{30}O_{16}$	-	-	-	717.1455	-0.9	519.0918,339.0503,3
27	16.960	salvianolic acid A[3][8]a	$C_{26}H_{22}O_{10}$	-	-	-	493.1132	-1.6	313.0722,295.0610,1
28	17.140	triptolide[10]a	$C_{20}H_{24}O_6$	361.164	0.6	361.1647,185.0970	-	-	-
29	17.596	wedelolactone [7]a	$C_{16}H_{10}O_7$	-	-	-	313.0350	-1.2	298.0112,270.0167
30	20.743	ecliptasaponin C[7]	$C_{42}H_{68}O_{14}$	-	-	-	841.4577	-1.7	795.4528,633.3996
31	24.255	wilfortrine[9]	$C_{41}H_{47}NO_{20}$	874.2769	0.5	856.2664,846.2810,176.0701	-	-	-

32	24.402	ecliptasaponin A[7]a	$C_{36}H_{58}O_9$			-	633.4002	-1	633.3991,587.3944
33	24.576	wilformine[9]	$C_{38}H_{47}NO_{18}$	806.2865	-0.1	788.2766,746.2659,686.2447	-	-	-
34	26.423	wilforgine [9-10]a	$C_{41}H_{47}NO_{19}$	858.2815	0	840.2721,686.2461,206.0814	-	-	-
35	26.757	dihydrotanshinone I[8]	$C_{18}H_{14}O_3$	279.1013	-1	261.0913,233.0961,190.0776	-	-	-
36	29.359	tanshinone Ia	$C_{18}H_{12}O_3$	277.086	0.2	249.0909,178.0780	-	-	-
37	31.818	tanshinone IIA[8]	$C_{19}H_{18}O_3$	295.1327	-0.7	277.1222,249.1278	-	-	-

A-*Tripterygium hypoglaucum (Levl.) Hutch*; B-*Eucommia ulmoides Oliver*; C-*Fructus Ligustri Lucidi*; D-*Fructus lycii*; E-*Herba ecliptae*; F-*Salvia miltiorrhiza Bge*; a-Comparison and identification of reference substances.

Reference

1. Wu YJ, Pang Lu, Li M, Li LX. Determination of betaine in Fructus Lycii by high performance liquid chromatography-electrospray detector [J]. Shandong Chemical Industry, 2018. 47 (12): 67-69.

2. Yan Y, Zhao H, Zou LS, Liu XH, Chai C, Wang SN, Hua YJ. LC-Triple TOF MS/MS analysis of chemical constituents of Eucommia ulmoides [J]. Journal of Mass Spectrometry, 2017. 38 (01): 146-156.

3. Gao T, Yang LJ, Sheng XK, Division BM, Han YQ, Jia P, Zhang YJ, Wang SX, Yu J, Zheng XH. Analysis of chemical constituents of Xiangdan injection based on HPLC-Q-TOF/MS and GC/M S [J]. Proprietary Chinese Medicine, 2019 and 41 (02): 345-352.

4. Yi J, Wu JG, Huang YX, Wu YB. Simultaneous determination of 8 main components in Ligustrum lucidum from different habitats by RP-HPLC. Fujian Chinese Medicine, 2017. 48 (06): 32-34.

5. Tan L, Dong Q, Xiao YC, Hu FZ. Simultaneous determination of five phenolic acids in Fructus Lycii by RP-HPLC. Journal of Drug Analysis, 20 130.33 (03): 376-394.

6. Xiao X, Xu S, Gao YM, Li S, Wu CR, Yan SK. Identification of Chemical constituents of Ligustrum lucidum by UPLC-Q/TOF MS [J]. The current generation of food science and technology, 2019jue 35 (10): 253-260.

7. Zhong XL, Wang RL, Duan L, Yu CQ, Xiao XR, Zhong YM. The material basis of synergism between Ligustrum lucidum and Ligustrum lucidum was analyzed based on UPLC/Q-TOF-MS technology [J]. Chinese Journal of Hospital Pharmacy, 2017 Journal 37 (19): 1887-1891.

8. Chen YQ, Fan XS, Zhu ZH, Peng GP, Duan JY. Analysis of chemical constituents of Shuangshen Pingfei granules based on UPLC-ESI-Q-TOF-MS/MS technique. Chinese herbal medicine, 2019 Personality 1-11.

9. Liu JQ, Russell, Zhang R, Shu JC, Yang RK, Yan J. Based on UPLC-Q-TOF MS, the effect of Tripterygium wilfordii on the chemical constituents of Tripterygium wilfordii was studied. Journal of Mass Spectrometry, 2018. 39 (05): 573-582.

10. Yang YG, Zhang Y, Weng DQ, Zhang Y. the contents of six active components in Tripterygium hypoglaucum from different producing areas and different parts were determined by UPLC-MS. Research and Development of Natural products, 2016 Magi 28 (03): 382-387.

11. Liu C, Hao QX, Jin Y, Huang LQ, Kang LP, Guo LP. Comparison of metabolic products between Tripterygium wilfordii and Tripterygium hypoglaucum leaves based on UPLC-Q-TOF-MS [J]. Chinese Journal of traditional Chinese Medicine, 2015 .40 (09): 1710-1717.
