# Supplementary material for: Real-world effects of Yishen Tongbi decoction for rheumatoid arthritis: protocol for a prospective, observational, multicenter cohort study with validation against double-blind, randomized, controlled trial
Source: Front Pharmacol. 2024 Feb 12;15:1320578. doi: 10.3389/fphar.2024.1320578 (PMC10895057; doi:10.3389/fphar.2024.1320578)
Supplement: Supplementary file 2 [file DataSheet3.PDF]

Table. 2. Adverse events of RCT study

Category	YSTB(n=49)	MTX(n=49)	Total(n=98)	P(between groups)
Drug-related	14(28.6)	11(22.4)	25(25.5)	0.487
Gastrointestinal	1(2.0)	1(2.0)	2(2.0)	1.000
Nausea and vomiting	0(0.0)	1(2.0)	1(1.0)	0.500
Diarrhoea	1(2.0)	0(0.0)	1(1.0)	0.500
Rash	1(2.0)	0(0.0)	1(1.0)	0.500
Palpitations	1(2.0)	0(0.0)	1(1.0)	0.500
ALT elevation	7(14.3)	6(12.2)	13(13.3)	0.766
Leucocytopenia	1(2.0)	1(2.0)	2(2.0)	1.000
Anemia	0(0.0)	1(2.0)	1(1.0)	0.500
Irregular menstruation	2(4.1)	1(2.0)	3(3.1)	0.554
