# Supplementary material for: Real-world effects of Yishen Tongbi decoction for rheumatoid arthritis: protocol for a prospective, observational, multicenter cohort study with validation against double-blind, randomized, controlled trial
Source: Front Pharmacol. 2024 Feb 12;15:1320578. doi: 10.3389/fphar.2024.1320578 (PMC10895057; doi:10.3389/fphar.2024.1320578)
Supplement: Supplementary file 3 [file DataSheet1.PDF]

Informed consent form for clinical research

Project name: application of Yishen Tongbi decoction in rheumatoid arthritis
Principal researcher: Chen Guangxing
Clinical trial institution: the first affiliated Hospital of Guangzhou University of traditional Chinese Medicine; Baiyun Hospital of The First Affiliated Hospital of Guangzhou University of Chinese Medicine
Version number: V2.0
Version date: September 5, 2023

Personal reading material

Dear patients, please read this article carefully. You are welcome to ask questions and discuss it with your family, relatives, friends or us.

You are invited to participate in this clinical study. The purpose of this study was to further evaluate the efficacy and safety of Yishen Tongbi decoction in the real-world treatment of rheumatoid arthritis.

Whether or not to participate in this study is entirely up to you. Please read this material carefully before you make a decision. It can help you to have a comprehensive understanding of the purpose, method, process, benefits and inconvenience of participating in the study, as well as your rights and interests. The information provided to you in this informed consent form can help you decide whether to participate in this clinical trial. If you have any questions, please ask the researcher in charge of the project trial, or discuss it with your family, relatives and friends. To ensure that you fully understand the relevant content. Whether you participate in this trial is voluntary. If you agree to participate in this clinical trial, please sign in the informed consent form.

1. What kind of research is this? [background and objective]

The name of this study is the application of Yishen Tongbi decoction in rheumatoid arthritis. The purpose of this study was to further evaluate the efficacy and safety of Yishen Tongbi decoction in the real-world treatment of rheumatoid arthritis. This study has been reviewed by the Ethics Committee of the first affiliated Hospital of Guangzhou University of traditional Chinese Medicine. It is considered that this study complies with the internationally recognized principles of the Helsinki Declaration and is in line with medical ethics.

2. Is it voluntary to participate in this study?

Your participation in this study is entirely voluntary. You have the right to decide whether or not to participate in this study, and you are not required to provide any reasons for your decision. Choosing not to participate in this study will not result in any discrimination or retaliation. It will have no impact on your relationship with your doctor or your medical care. You will continue to receive diagnosis and treatment from your doctor.

3. Who is not suitable to participate in this project?

Subject who does not meet any of the following will be excluded:
1)Pregnant or lactating women
2)Individuals with current or future fertility concerns

3)Currently using Chinese patent medicine or traditional Chinese medicine decoction that bear similarities to the experimental drugs in terms of their constituent components
4)History of chronic severe infection, any current infection and any malignant tumor
5)Patients with severe primary diseases, such as those of the cardiovascular, cerebrovascular, hepatic, renal, and hematopoietic systems, and mental illness

4. What are the drugs used in this study?

The drug selected in this study is Yishen Tongbi decoction.

The experimental medicine Yishen Tongbi decoction is provided by Kangmei Pharmaceutical Co., Ltd. It is composed of Tripterygium hypoglaucum (Levl.) Hutch, Eucommia ulmoides Oliver, Fructus Ligustri Lucidi, etc., which has the effect of tonifying kidney, expelling wind and promoting Qi flow, and is used in the treatment of active rheumatoid arthritis.

5. What will you need to do if you participate in the study?

If you participate in this study, you will enter the screening period after signing the informed consent form. You need to make the following checks to determine whether you can participate in this study:

If the doctor believes that you meet the selection criteria and are suitable for this study, you will decide how you will receive treatment according to your personal wishes. You can choose to join the Yishen Tongbi decoction combined with standard treatment group, or you can choose to enter the standard treatment group.

The total number of cases in this clinical study was 324, and the course of treatment was 12 weeks. The specific treatment plans of Yishen Tongbi decoction combined with standard treatment group and standard treatment group were as follows:

Yishen Tongbi decoction combined with standard treatment group: Yishen Tongbi decoction, 1 dose a day, 150ml, once a day, oral after meal; combined with conventional synthetic disease-modifying antirheumatic drugs (csDMARDs) to improve the condition, continuously taken for 12 weeks.

Standard treatment group: Biologic disease-modifying antirheumatic drugs (bDMARDs) / Targeted synthetic disease-modifying antirheumatic drugs (tsDMARDs) combined with csDMARDs, or a combination of two csDMARDs for 12 weeks.

There are no restrictions on the use of other types of drugs in the course of treatment, but records must be made.

Clinical evaluation and laboratory tests are required before the start of treatment, 4 weeks and 12 weeks of treatment to determine whether the treatment you are receiving is safe and effective. Clinical evaluation included pain joint number, swollen joint number, pain score, patient overall score, doctor overall score, clinical disease activity index (cDAI), disease activity index (DAS28), simplified disease activity index (sDAI) and so on. Laboratory tests included blood routine, ESR, C-reactive protein, liver, renal function and so on.

Please take the medicine according to the doctor's advice during the observation period. The doctor will tell you the time, method and revisit time in detail. During this period, you need to take other drugs, please consult your doctor first.

6. Possible benefits of participating in the research

You and society may or may not benefit directly from this study. Such benefits include the possibility that your condition may improve, and this study may help to further evaluate the efficacy and safety of Yishen Tongbi decoction in the treatment of rheumatoid arthritis in the real world, which is beneficial to the treatment of patients with similar conditions.

7. Possible adverse reactions, risks, discomfort and inconvenience to participate in the study

Any treatment may be ineffective, and the disease may continue to develop due to ineffective treatment or other diseases. This is the treatment risk that every patient will face, and even if he does not participate in this clinical study, the treatment risk will exist. During the study period, if the doctor or researcher finds that the treatment taken in this study is ineffective, the study will be terminated and other treatment measures that may be effective will be used.

Based on the data of previous clinical studies, a small number of patients taking Yishen Tongbi decoction may have transient increase of ALT, mild leukopenia, mild anemia or menstrual disorder, but can improve and relieve on their own.

8. Related fees

If you combine other diseases at the same time and have nothing to do with this study, the treatment and examination required, as well as the cost of switching to other treatments due to the suspension of the study, will not be free of charge. According to the relevant regulations, if any damage related to the research occurs, the sponsor will pay you the corresponding medical expenses and compensation.

9. How to protect your right to privacy?

Your medical records (including research medical records and physical and chemical examination reports, etc.) will be kept in the hospital as required. Your participation in the study and your personal data during the study will be kept confidential, and your personal identity will not be revealed in the report on the results of the study. Your superior health / drug / research administration, hospital ethics committee, researcher and sponsor representatives will be allowed to access your medical records in order to verify clinical research procedures and / or data. We will strictly protect the privacy of your personal medical data within the scope of the existing law.

10. Can I quit after taking part in the study?

Whether you participate or not depends entirely on your willingness. You can refuse to participate in this study or withdraw from the study at any time in the research process, without any reason, which will not affect your relationship with your doctor, nor will it affect the loss of your medical or other benefits. you will not be discriminated against or retaliated against.

Your doctor may suspend your participation in this study at any time in your best interest.

If you do not participate in this study, or drop out, there are many other alternative treatments. You do not have to participate in this study in order to treat your disease. If you withdraw from the study for any reason, you may be asked about your use of drugs in your best interest, and you may be required to undergo laboratory and

physical examinations if your doctor deems it necessary.

If you choose to participate in this study after full consideration, we hope you can persist in completing the whole research process.

11. Access to more information

You can ask any questions about this study at any time, and your doctor will leave you contact information so that you can answer your questions.

If there is any important new information during the study that may affect your willingness to continue to participate in the study, your doctor will inform you in a timely manner.

12. What to do now?

It is up to you to decide whether to participate in this study or not. You can also discuss it with your family before making a decision. Before you make the decision to participate in the study, please ask your doctor as much as possible until you fully understand the study.

Finally, thank you for reading the above materials. If you decide to participate in this study, please tell your doctor that they will arrange everything related to the research for you. Please keep this material. If you have any questions about your rights and interests in this study, please contact the Ethics Committee of the Centre at 020-36588667 or 020-36591965; email: gztcmlunli@163.com; Fax: 020-36591346.
